# The Prevalence of Liver Fibrosis Stages on More than 23,000 Liver Stiffness Measurements by Vibration-Controlled Transient Elastography: A Single Center Study

**DOI:** 10.3390/diagnostics13172803

**Published:** 2023-08-30

**Authors:** Alin Lazar, Ioan Sporea, Diana Lungeanu, Ruxandra Mare, Raluca Lupusoru, Alina Popescu, Mirela Danila, Alexandra Deleanu, Isabel Dan, Andrada Lascau, Alexandru Popa, Roxana Sirli

**Affiliations:** 1 Department of Internal Medicine II, Division of Gastroenterology and Hepatology, Center for Advanced Research in Gastroenterology and Hepatology, “Victor Babeș” University of Medicine and Pharmacy, 300041 Timișoara, Romania; lazar.alin@umft.ro (A.L.); isporea@umft.ro (I.S.); raluca.lupusoru@umft.ro (R.L.); popescu.alina@umft.ro (A.P.); danila.mirela@umft.ro (M.D.); deleanu.alexandra@umft.ro (A.D.); isabelstefan14@yahoo.com (I.D.); popa.alexandru@umft.ro (A.P.); sirli.roxana@umft.ro (R.S.); 2 Center for Modeling Biological Systems and Data Analysis, “Victor Babeș” University of Medicine and Pharmacy, 300041 Timișoara, Romania; dlungeanu@umft.ro; 3 Discipline of Accounting and Information System, Faculty of Economics and Business Administration, West University of Timișoara, 300115 Timișoara, Romania; andrada.lascau@gmail.com

**Keywords:** liver fibrosis, liver stiffness, liver diseases, non-invasive assessment

## Abstract

Vibration-controlled transient elastography (VCTE) was the first non-invasive method used for assessing liver fibrosis in patients with chronic liver disease. Over the years, many studies have evaluated its performance. It is now used globally, and, in some countries, it represents the primary step in evaluating liver fibrosis. The aim of this study is to assess the feasibility of VCTE and highlight the prevalence of liver fibrosis stages assessed by VCTE in a large cohort of patients at a single study center. We also aimed to observe the trends in liver stiffness (LS) values over the years according to each type of hepatopathy. A retrospective study was conducted over a period of 13 years (2007–2019) and included patients who presented to our clinic for LS measurements (LSMs), either with known liver diseases or with suspected liver pathology who were undergoing fibrosis screening. The database contained a total of 23,420 measurements. Valid LSMs were obtained in 90.91% (21,291/23,420) of the cases, while 2129 (9.09%) of the measurements were either failed or unreliable. In untreated patients with chronic viral hepatitis, LS values tended to increase during the years, while in patients undergoing antiviral therapy LS values significantly decreased. Our comprehensive study, one of the largest of its kind spanning 13 years, emphasizes the reliability and significance of VCTE in real-world clinical settings.

## 1. Introduction

According to the EFSUMB Guidelines, Ultrasound-based Shear Wave Elastography (SWE) techniques can be categorized into: Vibration Controlled Transient Elastography (FibroScan), Point SWE techniques, and SWE imaging (including 2D-SWE and 3D-SWE) [1]. Vibration Controlled Transient Elastography (VCTE) pioneered the non-invasive evaluation of liver fibrosis in patients afflicted with chronic liver diseases. Following its introduction, a multitude of ultrasound-based methodologies have been explored. Presently, manufacturers of ultrasonographic equipment are progressively advancing techniques specifically tailored to quantify hepatic stiffness as a non-invasive marker of fibrosis [2,3,4].

VCTE is a quantitative method that measures shear-wave speed using a surface impulse. The device comprises a piston embedded with the ultrasound transducer, which generates a mechanical “thump” into the liver which in turn generates shear waves that propagate perpendicular to the direction of the mechanical impulse. On the screen, an “M-mode” representation image displays axial displacement (brightness) as a function of depth and time. The shear-wave speed is determined by the decline of a straight line (white line), which best fits the depth dependence of a suitable reference point (termed “time of arrival”) [1]. Measurements with an incorrect vibration shape or without a correct follow-up of the vibration propagation are automatically rejected by the software.

The shear-wave speed can be converted to Young’s modulus value using the equation E = 3 ρc^2^s, with the measurement presented in kPa. This technique offers a regional elasticity measurement (spanning the width of the ultrasound beam and the depth of several centimeters for shear-wave penetration). However, it does not provide conventional ultrasound anatomical images, or 2D image guidance for the measurement, and cannot propagate the shear wave through ascites. In obese patients, it can be challenging to obtain sufficient signals using the standard M probe (3.5 MHz, 2 mm vibration amplitude). However, with the introduction of the XL probe (2.5 MHz, 3 mm vibration amplitude), the failure rate has decreased [4].

Over the years, VCTE performance has been analyzed in numerous studies. Currently, it is used globally and, in certain countries, it is the initial step for liver fibrosis evaluation [5,6,7]. VCTE is a fast, simple, reproducible non-invasive method used for assessing liver stiffness with well-defined quality criteria [6]. With a discriminating ability of >0.90, LSM by VCTE is still the most reliable method for identifying and ruling out advanced fibrosis and cirrhosis in all the major causes of chronic liver disease [6]. The convenience of repeatable assessments of hepatic stiffness during antiviral medication is one of the key advantages of non-invasive LSM. Repeated LSMs using VCTE in patients receiving antiviral therapy could help estimate the kinetics of liver stiffness decline over time and help determine the anticipated course during treatment because suppression of viral replication with antiviral therapy has been linked to a progressive decline in liver stiffness.

Recent advances in liver fibrosis detection have led to a plethora of non-invasive methodologies. While these methods offer promising results, the practical implications in terms of feasibility, global applicability, and consistency over a long period have not been extensively studied. Despite the influx of newer technologies, VCTE remains at the forefront due to its simplicity, speed, and reproducibility. The purpose of this study is to assess the feasibility of VCTE and to emphasize the prevalence of liver fibrosis stages as assessed by Vibration Controlled Transient Elastography (VCTE) in a vast real-world patient cohort from a single research center over an extended period. We also aimed to examine the trends in LSM values over the years based on different etiologies of chronic hepatopathies. This would provide clinicians and policymakers insights into its long-term reliability and potential as a primary assessment tool.

## 2. Materials and Methods

### 2.1. Study Population

A retrospective study was conducted over a period of about 13 years (2007–2019) and included patients who presented to our clinic for liver stiffness measurements (LSMs), either with known liver disease, but also patients with suspected liver pathology undergoing fibrosis screening. Patients under 18 years of age, with focal liver lesions, biliary obstruction, perihepatic ascites, pregnant women, patients with pacemakers, and patients with recent alcohol abuse were excluded. Furthermore, we included patients with repetitive LSM over the years.

When investigating the LSM values across time, three types of medical conditions were separately investigated: chronic viral hepatitis with hepatitis B virus (HBV), hepatitis C virus (HCV), HBV + HCV, HBV + HDV (hepatitis D virus), as well as chronic HBV HBe Antigen negative infection; autoimmune diseases (autoimmune hepatitis-AIH and primary biliary cholangitis—PBC); fatty liver diseases (including nonalcoholic fatty liver disease—NAFLD, nonalcoholic steatohepatitis—NASH, alcohol-associated liver disease—ALD, alcoholic steato-hepatitis—ASH, both alcoholic and non-alcoholic steatohepatitis—BASH). All patients signed a consent form to undergo LSMs using VCTE, which were performed by medical doctors experienced in this field (at least 100 LSMs performed for training).

### 2.2. Vibration Controlled Transient Elastography (VCTE)

Liver stiffness values were obtained using a model 502 FibroScan device (Echosens, Paris, France). The choice between the M or XL probe depended on each patient’s body mass index and skin-to-liver distance but in the first used version of FibroScan, the XL probe was unavailable. It only became available in 2012. For every patient, 10 measurements were performed in fasting conditions for at least 6 h; a median value was calculated and considered indicative of liver stiffness.

Transient elastography employs both M-mode and A-mode ultrasound imaging and the technique is performed by placing the probe in between the seventh and ninth intercostal space, perpendicular to the projection area of the right hepatic lobe, while the patient is in dorsal decubitus with his/her head resting on his/her right arm. The obtained values are expressed in kPa. A crucial reliability criterion for the median value of the 10 measurements is an Interquartile range to median ratio (IQR/M) of ≤30% [1]. A failed VCTE measurement was defined if no valid value was obtained after at least 10 shots, and a measurement was considered unreliable if fewer than 10 valid shots could be obtained and/or IQR/M ≥ 30% [1].

To discriminate between live fibrosis stages by VCTE we used the cut-offs proposed in the Tsochatzis meta-analysis: F2 ≥ 7 kPa; F3 ≥ 9.5 kPa and F4 ≥ 12 kPa [8].

### 2.3. Statistical Analysis

Descriptive statistics for categorical data, total frequency and percentages were used to illustrate the distribution of the relevant variables and their relationships to each other.

For investigating the LSM values across time, the repeated measures analysis of variance (ANOVA) in a general linear model (GLM) was employed, with the individual patients as the observational units, the sequence of measurements (e.g., first, second, etc., measurement) as a random crossover factor, and the medical condition and sex as nest factors. In order to yield the correct interaction effects, type III sums of squares were used to partition the unbalanced data variability and to determine the appropriate F statistics for the corresponding hypotheses of interest. Mauchly’s test for sphericity was applied and the Greenhouse–Geisser corrections were subsequently used for the ANOVA estimates. Estimated marginal means (EMMs) for two-by-two comparisons and contrasts between the nest factors’ levels (in parallel and across time) were determined using Tukey’s adjustments.

The statistical analysis was conducted at a 95% level of confidence and a 5% level of statistical significance. All reported probability values were two-tailed.

Analysis was performed using the statistical packages R 4.0.5 (supplementary including “ez” version 4.4-0, “afex” version 1.1-1, “emmeans” version 1.7.5, and “ggpubr” version 0.4.0).

## 3. Results

### 3.1. Determinants and Frequency of Unreliable and Failed Measurements

In total, the database contained 23,420 measurements. Out of these, valid LSMs (liver stiffness measurements) were obtained in 90.91% (21,291/23,420) of the measurements, and 2129 (9.09%) of the measurements were deemed failed or unreliable. LSMs were obtained by the M probe in 16,635 (71%) cases and by the XL probe in 6785 (29%) cases. 68.6% (1460/2129) of the invalid measurements were obtained by the M probe, and 31.4% (669/2129) were obtained using the XL probe. The distribution of unreliable/failed measurements according to the year of patient evaluation is summarized in Table 1.

The distribution of unreliable/failed measurements according to the etiology of the disease is summarized in Table 2.

The factors associated with unreliable or failed measurements were a BMI over 30 kg/m^2^, age over 60 years, male gender and metabolic disease (Table 3).

Patients with a BMI over 30 kg/m^2^ had 2.5 times the odds of unreliable LSMs in univariate analysis. However, when other factors were considered in multivariate analysis, this increased risk was reduced but remained significant. Older age (above 60 years) slightly increased the odds of unreliable LSMs, with minimal changes between univariate and multivariate analysis. Being male had a very slight increase in odds for unreliable LSMs in univariate analysis. However, in multivariate analysis, the odds ratio is slightly below 1, suggesting that when considering other factors, the male gender may not significantly increase the risk. Patients with metabolic disease had 1.5 times the odds of unreliable LSMs in univariate analysis, and this increased risk remained notable in multivariate analysis. All listed factors—high BMI, older age, male gender, and metabolic disease—have been associated with unreliable or failed LSMs to varying degrees but high BMI (over 30 kg/m^2^) and the presence of a metabolic disease show the most significant association with unreliable LSMs.

Based on VCTE cut-off values, the severity of liver fibrosis in our group of measurements (*n* = 21,291) was as follows: F < 2: 10,308 measurements (48.4%); F2: 3342 measurements (15.7%); F3: 1842 measurements (8.7%) and F4: 5799 measurements (27.2%). The distribution of measurements according to the severity of liver fibrosis and the year of the measurement is summarized in Table 4.

### 3.2. Characteristics of Study Population

After data cleansing and data curation, 8482 records remained, representing 2738 patients (44.6% men) with at least 3 LSM measurements. Descriptive statistics of patients regarding each etiology are presented in Supplementary Tables S1–S3.

### 3.3. Dynamics of LSMs over the Years

**Figure 1:**
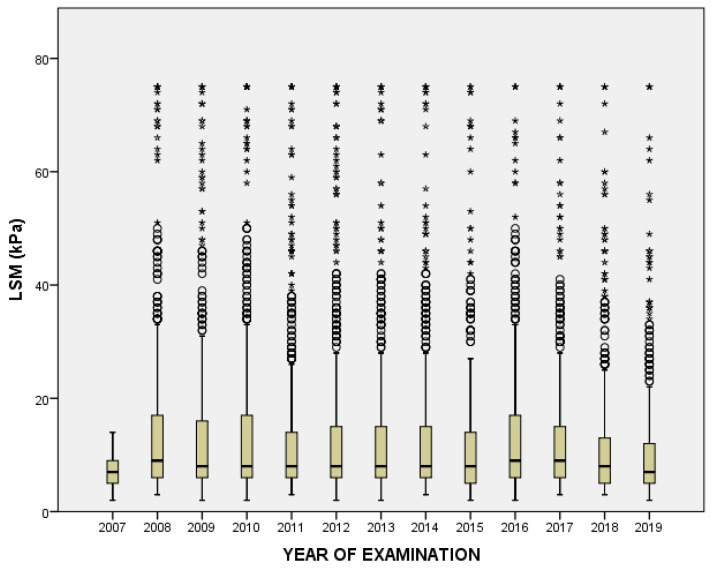
Shows the variability of liver stiffness measurements over the 13 years.

**Figure 2:**
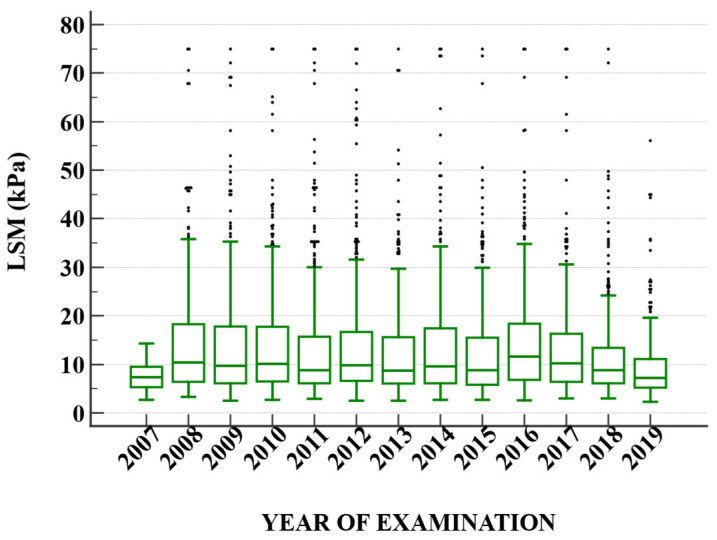
The dynamic of liver stiffness measurements in patients with hepatitis C virus infection. The dots represent the outliers.

**Figure 3:**
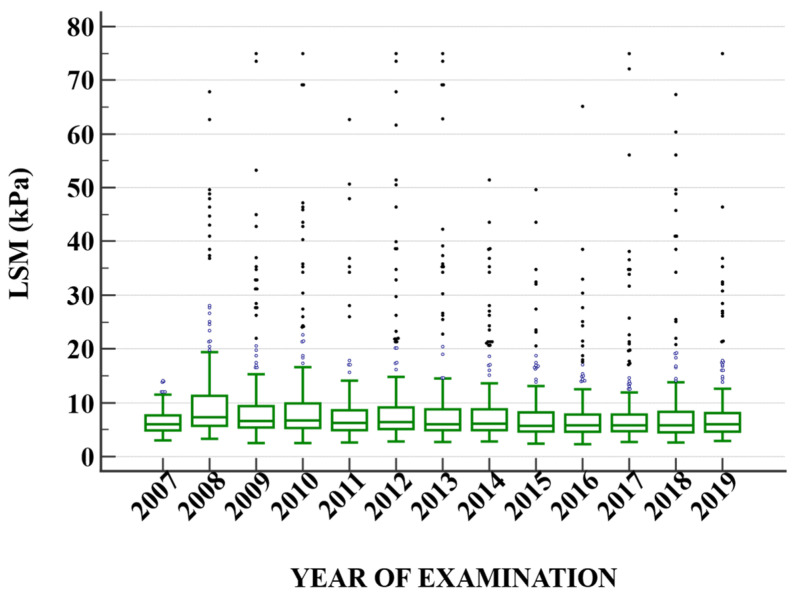
The dynamic of liver stiffness measurements in patients with hepatitis B virus infection.

Table 5 delineates the mean Liver Stiffness Measurement (LSM) values over a span of 13 years, from 2007 to 2019. The data reveal an upward spike in the mean LSM value in 2008, reaching 15.20, which is more than double the mean value of the previous year, 2007. This sudden increase is accompanied by a substantial standard deviation (SD) of 15.14, indicating a wide dispersion of the data points in 2008. Over the subsequent years, from 2009 to 2014, the mean LSM values stabilized within a range of approximately 13–14. However, starting from 2015, a gradual downward trend appears, with 2019 displaying the lowest mean value of 10.58 during the entire period (Figure 1). It is also noteworthy that while the mean values fluctuate across the years, the SD tends to decrease progressively, suggesting that the data points are becoming less dispersed and more concentrated around the mean as time progresses. When evaluating only HCV (Figure 2) and HBV (Figure 3) patients, respectively, we can observe a decrease over the years in HCV patients.

A subanalysis for HCV patients was performed. There was a significant difference between the patients treated with DAA where LSMs decreased in dynamics (16.55 ± 9.67 kPa vs. 11.12 ± 7.78 kPa, *p* = 0.02), while in non-treated HCV patients, there was a significant increase in LSMs dynamics (13.37 ± 10.62 kPa vs. 20.78 ± 12.13 kPa, *p* < 0.001) (Figure 2).

### 3.4. LSM Values according to Etiologies

When we analyzed the LSM values according to etiologies and gender, no matter how many measurements over time were performed in viral hepatitis patients, only the type of viral hepatitis and the masculine gender were associated with LSM values (Table 6 and Table 7).

**Figure 4:**
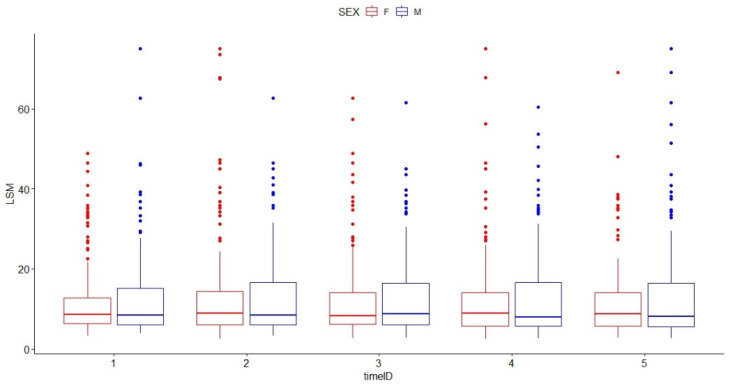
Boxplot graph of liver stiffness measurement values over time for the two sexes in patients with viral hepatitis (*n* = 326 viral hepatitis patients with at least five liver stiffness measurement values). The dots represent the outliers. The outliers for femeles are presented in Red and for males in Blue.

Table 8 and Table 9 and Figure 4 show the estimated mean values of LSM according to gender and number of LSM values over time.

When we analyzed the LSM values according to AID, we can see that no matter the autoimmune disease type, or the gender of time, none of the factors associated with the LSM value (Table 10). The estimated LSM mean values are shown in Table 11.

For fatty liver disease, the factors associated with the LSM value were the type of FLD (Table 12). The estimated LSM mean values are showed in Table 13.

## 4. Discussion

Vibration Controlled Transient Elastography is the most widely used non-invasive method for the evaluation of liver stiffness in chronic liver diseases and appears to be the most accurate non-invasive approach for early identification of cirrhosis [1,9,10].

With immediate results and excellent patient acceptance, VCTE is a user-friendly technique that can be quickly conducted (at bedside) and has become a crucial tool in clinical practice for assessing liver fibrosis [7]. To our knowledge, this is the largest study that performed liver stiffness measurements using VCTE over a period of 13 years on more than 23,000 LSMs.

Despite its primarily descriptive nature, the large amount of the dataset warranted such an approach for effective summarization. From the 23,420 measurements, the feasibility of VCTE measurements by M and XL probes was 91.2%.

There has been a general decline in the percentage of failed and unreliable LSMs over the years. The data show that the highest percentage of failed and unreliable measurements occurred around 2012, but by 2019, this was reduced to the lowest percentage in the presented data, suggesting improvements in the reliability of the measurements over time. 2012 had the highest percentage of unreliable LSMs at 12.45%, equating to 265 unreliable measurements. Post-2012, there was a noticeable decline in the percentage of unreliable LSMs, with the most significant drop happening between 2014 and 2015. This might indicate technological advancements, better training, or other interventions that increased the reliability of the measurements. From prior information, we observed that the M probe accounted for a more significant portion of the unreliable and failed measurements compared to the XL probe, a known drawback of the M probe being the inability to obtain valid LSM in patients with high BMI. Another factor should be taken into consideration, the XL probe has been available only since 2012.

Similarly, with other studies, the majority of invalid/unreliable LSM were obtained with the M probe, in direct relation to the patients’ BMI [11,12]. Our study indicated a significant decrease in the failure rate of VCTE measurements over the years. While we suggested the introduction of the XL probe as one potential reason, another plausible explanation is the increased proficiency of operators during time, as clinicians become more adept with the technology, and therefore, the reliability of measurements has likely improved. Continuous training and workshops have potentially contributed to this trend. This highlights the dual importance of embracing technological advancements and ensuring that medical practitioners are adequately trained to leverage these tools effectively.

The majority of our patients with unreliable or failed LSM were HCV patients (34.5%) representing also the majority of patients with repeated measurements across the 13-year periods of time, followed by NAFLD patients (22.4%). One explanation for the high number of unreliable or failed measurements in patients with NAFLD might be the higher BMI. In the absence of a potent treatment other than physical activity and diet in patients with NALFD, over the years we have seen that a minority of patients lost weight. The factors found involved in the unreliable results are BMI, age, gender and metabolic disease.

Table 2 with the etiology underscores the high prevalence of viral hepatitis, particularly HCV and HBV, among the patient population studied. These two conditions combined account for over 58% of all measurements, considering that we are a tertiary hepatology center, implicated in the diagnosis and treatment of chronic viral hepatitis. Metabolic liver diseases, such as NASH and NAFLD, are also significant contributors, representing the third most common etiology. Some etiologies are relatively rare, indicating that they are either less common in the general population or possibly underrepresented in the sample. A comprehensive assessment like this helps to understand the distribution and burden of various liver diseases in a specific patient population or setting. It also provides insights into the challenges and priorities for healthcare providers in managing and preventing these conditions.

Furthermore, we evaluated the kinetics of repeated LSM over time in patients with chronic viral hepatitis (HBV, HCV, HBV + HCV, HBV + HDV, as well as in HBV HBe Ag negative chronic infection); autoimmune diseases (AIH or PBC); fatty liver diseases (NAFLD, NASH, ALD, ASH, BASH). Out of 1374 patients with chronic viral liver diseases, 1048 had at least three LSM performed over time, while the remaining had five LSM. We chose to analyze both groups (3 vs. 5 LSM over 13 years) because the longer the duration, the greater the chance of having a correct modeling of reality based on the collected and processed data. At the same time, more patients (e.g., 1084 patients with three readings compared to 326 with five readings) make the standard errors smaller and the statistical power increase. Either way, time does not seem to be a decisive element in the dynamics of LSM. One possible explanation is that in the overall analysis of our cohort, we did not divide our patients into two subgroups (under treatment vs. no treatment). LSM values are strongly dependent on the type of infection in viral liver diseases. In the majority of cases of HBV infections, patients have high levels of transaminases whereas in HCV patients the transaminases can be normal.

On the other hand, there is a clear relationship between time periods and LSM in patients with autoimmune hepatopathies and those with fatty liver of different causes. A decrease in the variability of LSM values over time in patients with alcoholic liver disease (ALD) was seen; a fact that can be linked to the patient’s abstinence from alcohol.

When analyzing separately HBV patients undergoing antiviral therapy we observed a progressive decline in liver stiffness, particularly in patients with high baseline alanine aminotransferase and viral load. The same observation was made in HCV patients following antiviral treatment with DAA in whom LSM significantly decreased, results similar to previously published papers [13,14,15,16,17].

Upon examining the HBV cohort, we observed a consistent increase in liver stiffness up to the point of intervention with treatment. This trend aligns well with existing literature on the subject [18,19].

Our study has some limitations. First, due to its retrospective nature and long follow-up period, patients have been evaluated for liver fibrosis only using a non-invasive method which was VCTE and not by using liver biopsy. However, it is difficult to conduct serial biopsies in large cohorts for follow-up in clinical practice. Another limitation of the study was the heterogeneity of patients, variability in etiologies and treatment for different liver diseases and most importantly the variability of LSM at different time periods.

Despite these limitations, our extensive 13-year retrospective analysis provides a comprehensive understanding of its effectiveness. It further dissects the trends in liver stiffness across various hepatopathies, offering a granular insight into its relevance across diverse patient groups. This work stands as a testament to the sustainability of VCTE as a non-invasive assessment tool, even in the face of emerging technologies.

The vast utilization of Vibration Controlled Transient Elastography (VCTE) for liver stiffness measurement signifies the profound paradigm shift in how clinicians approach liver disease assessment. Previously, invasive procedures such as liver biopsy were the gold standard, but they came with potential complications and patient discomfort. VCTE, being non-invasive, offers a safer alternative, reducing the risks associated with biopsies and increasing patient compliance. Due to the procedure’s safety, non-invasiveness, and excellent reproducibility, the American Gastroenterological Association (AGA) technical study [20] strongly suggests adopting VCTE to replace liver biopsy in people with HCV. Our extensive data support the notion that VCTE not only offers a diagnostic solution but also provides a reliable tool for monitoring disease progression over extended periods. The growing reliance on VCTE in clinical practice allows for earlier detection of fibrosis and cirrhosis, which is paramount for timely interventions and improved patient outcomes.

## 5. Conclusions

This comprehensive study, one of the largest of its kind spanning 13 years, emphasizes the reliability and significance of VCTE in real-world clinical settings, with a feasibility of more than 90%. Furthermore, the study provides an in-depth analysis of liver stiffness variations among different liver diseases, shedding light on its significance for a wide range of patient groups.

## Figures and Tables

**Table 1:** The distribution of unreliable/failed measurements according to the year of evaluation.

Year of Evaluation	Unreliable LSMs *n* = 2129 Percentage (Number of Measurements)
2008	7.8% (166)
2009	9.35% (199)
2010	11.09% (236)
2011	7.8% (166)
2012	12.45% (265)
2013	11.2% (239)
2014	11.7% (250)
2015	5.1% (108)
2016	7.3% (155)
2017	6.2% (133)
2018	5.7% (122)
2019	4.2% (90)

Note: *n* = number; LSMs = liver stiffness measurements.

**Table 2:** The distribution of unreliable/failed measurements according to the etiology of the disease.

Etiology of Liver Disease		Measurements with Unreliable/Failed LSMs *n* = 2129 Percentage (Number of Cases)
ALD		6.1% (130)
BASH		0.2% (5)
HBV		17.8% (379)
HCV		34.5% (734)
Mixed etiology	HBV and ALD	0.05% (1)
	HCV and ALD	0.05% (1)
	HBV and HCV	0.8% (17)
	HBV and HDV	0.9% (19)
Autoimmune hepatitis		1.5% (32)
NASH/NAFLD		22.4% (476)
PBC		1.5% (32)
Chronic hepatitis		6.6% (140)
Cryptogenic cirrhosis		7% (150)
Portal hypertension		0.09% (2)
Normal		0.6% (12)

Note: *n* = number; ALD = alcoholic liver disease, BASH = Both Alcoholic and Non-Alcoholic Steatohepatitis, HBV = hepatitis B virus infection, HCV = hepatitis C virus infection, HDV = hepatitis D virus infection, NASH = Non-Alcoholic Steatohepatitis; NAFLD = Non-alcoholic fatty liver disease, PBC = primary biliary cholangitis, Normal = measurements from subjects without known liver disease.

**Table 3:** Analysis of factors associated with unreliable/failed measurements.

Parameter	Univariate Analysis			Multivariate Analysis		
	OR	95% CI	*p* Value	OR	95% CI	*p* Value
BMI (>30 kg/m^2^)	2.5	1.52–2.95	<0.0001	1.16	1.02–1.54	<0.0001
Age (>60 years)	1.03	1.00–1.05	0.001	1.00	1.00–1.25	0.01
Male gender	1.01	0.98–1.05	0.04	0.99	0.99–1.01	0.01
Metabolic disease *	1.50	1.10–2.10	0.0001	1.10	1.00–1.80	0.0001

* Three out of five criteria of the metabolic syndrome being met.

**Table 4:** The distribution of measurements according to the severity of liver fibrosis and the year of patient evaluation.

Year of Examination	Severity of Liver Fibrosis			
	F0–F1 *n* = 10,308	F2 *n* = 3342	F3 *n* = 1842	F4 *n* = 5799
2007	3.8% (392)	3.9% (131)	4.39% (81)	0.6% (36)
2008	8.5% (874)	8.2% (274)	8.2% (151)	11.6% (672)
2009	7.4% (766)	7.9% (265)	8% (147)	8.5% (495)
2010	6.2% (640)	7% (234)	7% (129)	7.4% (429)
2011	6.5% (669)	6.6% (221)	6.9% (127)	7.1% (411)
2012	7.8% (805)	8.2% (273)	7.7% (141)	7.4% (429)
2013	7.9% (816)	7.2% (242)	7.7% (141)	7.7% (446)
2014	9% (928)	8.6% (289)	8.9% (164)	8% (465)
2015	6.7% (691)	6% (202)	5.8% (107)	6% (349)
2016	8.9% (913)	8.4% (282)	7.8% (143)	11% (640)
2017	9.3% (955)	8.3% (276)	10.3% (190)	9.5% (553)
2018	9.5% (975)	10.9% (365)	10% (184)	8.9% (514)
2019	8.6% (884)	8.6% (287)	7.4% (137)	6% (350)

Note: The percentages refer to the distribution of each diagnosis over the years. The VCTE-Cutoff was set as follows: F0–F1 < 7 kPa; F2 ≥ 7 kPa; F3 ≥ 9.5 kPa and F4 ≥ 12 kPa.

**Table 5:** LSM mean values for the 13 years.

Parameter	Mean	SD
(1) 2007	7.18	2.73
(2) 2008	15.20	15.14
(3) 2009	14.03	14.05
(4) 2010	14.52	14.73
(5) 2011	13.61	14.08
(6) 2012	14.28	14.58
(7) 2013	13.20	13.13
(8) 2014	13.04	12.62
(9) 2015	11.89	11.71
(10) 2016	13.07	11.31
(11) 2017	12.55	11.38
(12) 2018	11.35	10.26
(13) 2019	10.58	10.21

**Table 6:** Repeated measures ANOVA for the patients with viral hepatitis (*n* = 326 VH patients with at least five LSM values).

Outcome: LSM Value			
Effect	df with GG Correction	F	*p*-Value
VH	4, 320	5.75	<0.001 **
Sex	1, 320	8.31	0.004 **
timeID	3.40, 1087.31	0.69	0.576
VH:timeID	13.59, 1087.31	0.50	0.931
Sex:timeID	3.40, 1087.31	1.41	0.236

Statistical significance: **, *p* < 0.01. Abbreviations: df, degrees of freedom; GG, Greenhouse–Geisser correction for departure from sphericity; VH, type of viral hepatitis; LSM, liver stiffness measurement; timeID, LSM measurements’ sequence.

**Table 7:** Repeated measures ANOVA for the patients with viral hepatitis (*n* = 1084 VH patients with at least three LSM values).

Outcome: LSM Value			
Effect	df with GG Correction	F	*p*-Value
VH	4, 1078	21.31	<0.001 **
Sex	1, 1078	17.22	<0.001 **
timeID	1.93, 2075.90	2.84	0.061 +
VH:timeID	7.70, 2075.90	0.96	0.460
Sex:timeID	1.93, 2075.90	0.47	0.619

Statistical significance: **, *p* < 0.01; +, *p* < 0.1. Abbreviations: df, degrees of freedom; GG, Greenhouse–Geisser correction for departure from sphericity; VH, type of viral hepatitis; LSM, liver stiffness measurement; timeID, LSM measurements’ sequence.

**Table 8:** Estimated marginal means for the patients with viral hepatitis (*n* = 1084 VH patients with at least three LSM values). Values were averaged over the levels of timeID and Tukey’s adjustments were applied for a family of 10 estimates.

Outcome: LSM Value			
VH	Sex M	Estimated Mean ± SE	df
HBV Hbe Ag negative infection	0	3.90 ± 1.240	1078
HBV	0	7.70 ± 0.681	1078
HCV	0	12.42 ± 0.393	1078
HBV + HDV	0	10.54 ± 3.258	1078
HCV + HBV	0	14.60 ± 1.896	1078
HBV Hbe Ag negative infection	1	6.33 ± 1.249	1078
HBV	1	10.12 ± 0.621	1078
HCV	1	14.85 ± 0.513	1078
HBV + HDV	1	12.97 ± 3.297	1078
HCV + HBV	1	17.03 ± 1.911	1078

Abbreviations: df, degrees of freedom; VH, type of viral hepatitis; LSM, liver stiffness measurement; timeID, LSM measurements’ sequence; SE, standard error of the estimate.

**Table 9:** Estimated marginal means for the patients with viral hepatitis (*n* = 326 VH patients with at least five LSM values). Values were averaged over the levels of timeID and Tukey’s adjustments were applied for a family of 10 estimates.

Outcome: LSM Value			
HV	Sex M	Estimated Mean ± SE	df
HBV Hbe Ag negative infection	0	2.43 ± 2.64	320
HBV	0	8.13 ± 1.38	320
HCV	0	12.62 ± 0.70	320
HBV + HDV	0	12.91 ± 4.70	320
HCV + HBV	0	15.05 ± 3.84	320
HBV Hbe Ag negative infection	1	5.74 ± 2.53	320
HBV	1	11.44 ± 1.17	320
HCV	1	15.93 ± 1.02	320
HBV + HDV	1	16.22 ± 4.77	320
HCV + HBV	1	18.35 ± 3.95	320

Abbreviations: df, degrees of freedom; VH, type of viral hepatitis; LSM, liver stiffness measurement; timeID, LSM measurements’ sequence; SE, standard error of the estimate.

**Table 10:** Repeated measures ANOVA for the patients with autoimmune disease (*n* = 45 AID patients with at least three LSM values).

Outcome: LSM Value			
Effect	df with GG Correction	F	*p*-Value
AID	1, 42	0.54	0.465
Sex	1, 42	1.10	0.301
timeID	1.89, 79.27	0.22	0.794
AID:timeID	1.89, 79.27	3.22	0.048 *
Sex:timeID	1.89, 79.27	0.55	0.571

Statistical significance: *, *p* < 0.05. Abbreviations: AID, type of autoimmune disease (AIH or PBC); df, degrees of freedom; GG, Greenhouse-Geisser correction for departure from sphericity; LSM, liver stiffness measurement; timeID, LSM measurements’ sequence.

**Table 11:** Estimated marginal means for the patients with autoimmune disease (*n* = 45 AID patients with at least three LSM values). Values were averaged over the levels of timeID and Tukey’s adjustments were applied for a family of 4 estimates.

Outcome: LSM Value			
AID	Sex M	Estimated Mean ± SE	df
AIH	0	14.26 ± 2.93	42
PBC	0	11.58 ± 2.36	42
AIH	1	8.78 ± 5.18	42
PBC	1	6.11 ± 5.18	42

Abbreviations: AID, type of autoimmune disease; df, degrees of freedom; LSM, liver stiffness measurement; timeID, LSM measurements’ sequence; SE, standard error of the estimate; PBC = primary biliary cholangitis; AIH = autoimmune hepatitis.

**Table 12:** Repeated measures ANOVA for the patients with fatty liver disease (*n* = 175 FLD patients with at least three LSM values).

Outcome: LSM Value			
Effect	df with GG Correction	F	*p*-Value
FLD	4, 169	39.81	<0.001 **
Sex	1, 169	0.02	0.895
timeID	1.63, 276.19	1.08	0.331
FLD:timeID	6.54, 276.19	3.62	0.001 **
Sex:timeID	1.63, 276.19	1.13	0.316

Statistical significance: **, *p* < 0.01. Abbreviations: df, degrees of freedom; FLD, type of fatty liver disease; GG, Greenhouse–Geisser correction for departure from sphericity; LSM, liver stiffness measurement; timeID, LSM measurements’ sequence.

**Table 13:** Estimated marginal means for the patients with fatty liver disease (*n* = 175 FLD patients with at least three LSM values). Values were averaged over the levels of timeID and Tukey’s adjustments were applied for a family of 10 estimates.

Outcome: LSM Value			
FLD	Sex M	Estimated Mean ± SE	df
NAFLD	0	7.77 ± 2.26	169
NASH	0	11.07 ± 1.68	169
ALD	0	33.17 ± 2.28	169
ASH	0	10.27 ± 4.81	169
BASH	0	7.84 ± 4.64	169
NAFLD	1	7.50 ± 2.26	169
NASH	1	10.80 ± 1.75	169
ALD	1	32.90 ± 1.35	169
ASH	1	10.01 ± 4.52	169
BASH	1	7.57 ± 4.17	169

Abbreviations: df, degrees of freedom; FLD, type of fatty liver disease; LSM, liver stiffness measurement; timeID, LSM measurements’ sequence; SE, standard error of the estimate; ALD = alcoholic liver disease, ASH = alcoholic steatohepatitis, BASH = Both Alcoholic and Non-Alcoholic Steatohepatitis, NASH = Non-Alcoholic Steatohepatitis; NAFLD = Non-alcoholic fatty liver disease.

## Data Availability

Not applicable.

## Supplementary Materials

The following supporting information can be downloaded at: https://www.mdpi.com/article/10.3390/diagnostics13172803/s1, Table S1: Descriptive statistics for patients with viral hepatitis. For each year, the number of enrolled patients is specified, with their age at enrollment and the number of LSM values recorded across the analyzed 13-year period. Table S2: Descriptive statistics for patients with autoimmune disease. For each year, the number of enrolled patients is specified, with their age at enrollment and the number of LSM values recorded across the analyzed 13-year period. Table S3: Descriptive statistics for patients with fatty liver disease. For each year, the number of enrolled patients is specified, with their age at enrollment and the number of LSM values recorded across the analyzed 13-year period.
