# Supplementary material for: The Prevalence of Liver Fibrosis Stages on More than 23,000 Liver Stiffness Measurements by Vibration-Controlled Transient Elastography: A Single Center Study
Source: Diagnostics (Basel). 2023 Aug 30;13(17):2803. doi: 10.3390/diagnostics13172803 (PMC10486787; doi:10.3390/diagnostics13172803)
Supplement: Supplementary file 1 [file diagnostics-13-02803-s001.zip › diagnostics-2570657-supplementary.pdf]

Supplimentary table S1: Descriptive statistics for patients with viral hepatitis. For each year, the number of enrolled patients is specified, with their age at enrollment and the number of LSM values recorded across the analyzed 13-year period

Number of patients	Total N=2054	HBV inactive N=134	HBV N=584	HCV N=1275	HBV + HCV N=19	HBV + HDV N=42
2007: N enrolled	214	20	44	142	3	5
Number of LSMs[(a)]	4 (2–5)	3 (2–4.5)	2.5 (2–4)	3 (2–5)	3 (2.5–6)	4 (2–4)
Age in years[(a)]	52 (44–60)	50 (36–54)	46 (33–54)	55 (49–64)	49 (47–50)	44 (43–52)
Sex M[(b)]	85 (39.7%)	13 (65%)	24 (54.5%)	44 (31%)	1 (33.3%)	3 (60%)
2008: N enrolled	360	26	92	223	6	13
Number of LSMs[(a)]	3 (2–5)	2.5 (2–4)	3 (2–5)	2 (2–3)	2 (2–2)	2 (2–3)
Age in years[(a)]	53 (43–59)	36.5 (26–47)	47 (31–54.5)	55 (49–61)	57 (50–64)	51 (46–54)
Sex M[(b)]	172 (47.8%)	13 (50%)	62 (67.4%)	88 (39.5%)	1 (16.7%)	8 (61.5%)
2009: N enrolled	278	19	79	167	6	7
Number of LSMs[(a)]	3 (2–4)	2 (2–2.5)	2 (2–3.5)	3 (2–4)	3 (2–6)	3 (2–5)
Age in years[(a)]	52 (43–58)	48 (33–53)	46 (36–55)	53 (47–60)	58 (49–74)	52 (52–53.5)
Sex M[(b)]	108 (38.8%)	12 (63.2%)	46 (58.2%)	46 (27.5%)	3 (50.0%)	1 (14.3%)
2010: N enrolled	183	19	50	108	1	5
Number of LSMs[(a)]	3 (2–4)	3 (2–4)	2 (2–3)	3 (2–4)	3	3 (2–3)
Age in years[(a)]	51 (42–57)	37 (30–51)	43.5 (30–53)	53 (47–60)	65	56 (51–57)
Sex M[(b)]	80 (43.7%)	9 (47.4%)	28 (56.0%)	40 (37.0%)	–	3 (60.0%)
2011: N enrolled	164	13	48	97	1	5

Number of LSMs[(a)]	3 (2–4)	2 (2–3)	3 (2–3)	3 (2–4)	2	3 (2–4)
Age in years[(a)]	54 (43.5–60)	45 (32–54)	52 (33–56.5)	56 (49–63)	57	49 (45–53)
Sex M[(b)]	68 (41.5%)	6 (46.2%)	25 (52.1%)	35 (36.1%)	–	2 (40.0%)
2012: N enrolled	150	8	42	97	–	3
Number of LSMs[(a)]	3 (2–4)	2 (2–2.5)	3 (2–4)	3 (2–4)	–	3 (2.5–4)
Age in years[(a)]	54 (45–59)	42.5 (25.5–61)	49 (34–54)	56 (50–61)	–	59 (56–60.5)
Sex M[(b)]	68 (45.3%)	6 (75.0%)	26 (61.9%)	35 (36.1%)	–	1 (33.3%)
2013: N enrolled	133	5	46	81	1	–
Number of LSMs[(a)]	2 (2–4)	2 (2–4)	2 (2–3)	2 (2–4)	2	–
Age in years[(a)]	55 (47–62)	36 (27–38)	52 (35–58)	57 (54–64)	33	–
Sex M[(b)]	53 (39.8%)	2 (40.0%)	25 (54.3%)	25 (30.9%)	1 (100%)	–
2014: N enrolled	121	3	48	67	1	2
Number of LSMs[(a)]	2 (2–3)	2 (2–2.5)	2 (2–3)	2 (2–3)	2	3 (2–4)
Age in years[(a)]	56 (42–63)	38 (31.5–41)	45.5 (35–56)	61 (52.50–67)	66	57 (53–61)
Sex M[(b)]	44 (36.4%)	2 (66.7%)	23 (47.9%)	18 (26.9%)	–	1 (50%)
2015: N enrolled	88	7	26	53	–	2
Number of LSMs[(a)]	3 (2–4)	3 (2–3)	2 (2–3)	3 (2–4)	–	3.5 (3–4)
Age in years[(a)]	54 (45.5–62)	57 (42.5–63)	46.5 (38–54)	58 (51–65)	–	57.5 (54–61)
Sex M[(b)]	29 (39.9%)	4 (57.1%)	10 (38.5%)	15 (28.3%)	–	–

2016: N enrolled	153	8	41	104	–	–
Number of LSMs[(a)]	2 (2–3)	2 (2–3)	2 (2–2)	3 (2–3)	–	–
Age in years[(a)]	58 (50–64)	55.5 (42–66)	49 (36–59)	60 (55–66)	–	–
Sex M[(b)]	62 (40.5%)	2 (25.0%)	22 (53.7%)	38 (36.5%)	–	–
2017: N enrolled	112	3	38	71	–	–
Number of LSMs[(a)]	2 (2–3)	2	2 (2–2)	3 (2–3)	–	–
Age in years[(a)]	61 (51.5–67)	61 (58.5–63.5)	53 (44–58)	64 (59–70.5)	–	–
Sex M[(b)]	43 (38.4%)	–	19 (50.0%)	24 (33.8%)	–	–
2018: N enrolled	96	3	30	63	–	–
Number of LSMs[(a)]	2 (2–3)	2	2	3 (2–3)	–	–
Age in years[(a)]	60 (51.5–66.5)	35 (35–51.5)	58 (36–64)	60 (56–68)	–	–
Sex M[(b)]	36 (37.5%)	1 (33.3%)	14 (46.7%)	21 (33.3%)	–	–
2019: N enrolled	2	–	–	2	–	–
Number of LSMs[(a)]	2	–	–	2	–	–
Age in years[(a)]	63	–	–	63	–	–
Sex M[(b)]	–	–	–	–	–	–

[(a)] either median (IQR) or the value when it was constant; [(b)] N (%)

Supplimentary table S2: Descriptive statistics for patients with autoimmune disease. For each year, the number of enrolled patients is specified, with their

age at enrollment and the number of LSM values recorded across the analyzed 13-year period

Number of patients	Total<br>N=82	AIH<br>N=29	PBC<br>N=53
**2007**: N enrolled	9	4	5
Number of LSMs[(a)]	4 (2–5)	5 (3–7.5)	3 (2–4)
Age in years[(a)]	55 (51–60)	56 (53–58.5)	55 (51–61)
Sex M[(b)]	1 (11.1%)	1 (25%)	–
**2008**: N enrolled	23	6	17
Number of LSMs[(a)]	4 (2–5)	3 (2–4)	5 (2–5)
Age in years[(a)]	52 (44.5–58.5)	53 (47–60)	52 (42–57)
Sex M[(b)]	1 (4.3%)	–	1 (5.9%)
**2009**: N enrolled	13	5	8
Number of LSMs[(a)]	3 (2–5)	4 (3–5)	2 (2–4.5)
Age in years[(a)]	52 (43–54)	54 (53–56)	46.5 (30.5–51.5)
Sex M[(b)]	1 (7.7%)	1 (20.0%)	–
**2010**: N enrolled	7	1	6
Number of LSMs[(a)]	5 (2–5)	2	5 (2–5)
Age in years[(a)]	55 (52–63.5)	68	54 (52–59)
Sex M[(b)]	2 (28.6%)	–	2 (33.3%)
**2011**: N enrolled	8	3	5
Number of LSMs[(a)]	3 (2–4)	3 (3–3.5)	3 (2–4)
Age in years[(a)]	57.5 (47–63.5)	64 (63.5–68)	55 (39–55)
Sex M[(b)]	2 (25.0%)	1 (33.3%)	1 (20.0%)
**2012**: N enrolled	2	2	–
Number of LSMs[(a)]	5 (3–7)	5 (3–7)	–
Age in years[(a)]	46.5 (37–56)	46.5 (37–56)	–
Sex M[(b)]	–	–	–
**2013**: N enrolled	7	3	4

Number of LSMs[(a)]	3 (2–3)	3 (2.5–3)	2.5 (2–4)
Age in years[(a)]	54 (49–54)	54 (49.5–54)	53.5 (39.5–60.5)
Sex M[(b)]	1 (14.3%)	–	1 (25.0%)
**2014**: N enrolled	3	2	1
Number of LSMs[(a)]	2 (2–3)	2	2
Age in years[(a)]	58 (38–65)	38 (18–58)	72
Sex M[(b)]	2 (66.7%)	1 (50.0%)	1 (100%)
**2015**: N enrolled	1	–	1
Number of LSMs[(a)]	2	–	2
Age in years[(a)]	62	–	62
Sex M[(b)]	–	–	–
**2016**: N enrolled	4	3	1
Number of LSMs[(a)]	2 (2–3)	2 (2–3)	2
Age in years[(a)]	56 (35–70)	70 (56–70)	28
Sex M[(b)]	1 (25%)	1 (33.3%)	–
**2017**: N enrolled	1	–	1
Number of LSMs[(a)]	3	–	3
Age in years[(a)]	47	–	47
Sex M[(b)]	–	–	–
**2018**: N enrolled	4	–	4
Number of LSMs[(a)]	2	–	2
Age in years[(a)]	56 (52.5–60.5)	–	56 (52.5–60.5)
Sex M[(b)]	–	–	–
**2019**: N enrolled	–	–	–

[(a)] either median (IQR) or the value when it was constant; [(b)] N (%)

Supplimentary table S3: Descriptive statistics for patients with fatty liver disease. For each year, the number of enrolled patients is specified, with their age at enrollment and the number of LSM values recorded across the analyzed 13-year period.

Number of patients	Total N=532	NAFLD N=136	NASH N=186	ALD N=165	ASH N=32	BASH N=13
2007: N enrolled	23	6	9	1	7	–
Number of LSMs[(a)]	2 (2–3)	2.5 (2–4)	2 (2–3)	3	2 (2–2)	–
Age in years[(a)]	51 (38–53)	49.5 (48–52)	38 (27–52)	44	54 (51–58.5)	–
Sex M[(b)]	16 (69.6%)	2 (33.3%	7 (77.8%)	–	7 (100%)	–
2008: N enrolled	61	8	25	22	5	1
Number of LSMs[(a)]	2 (2–3)	2 (2–2.5)	2 (2–3)	2 (2–3)	2 (2–2)	3
Age in years[(a)]	53 (44–59)	44.5 (35–53.5)	50 (41–55)	57 (52–60)	51 (49–58)	35
Sex M[(b)]	48 (78.7%)	6 (75.0%)	17 (68.0%)	19 (86.4%)	5 (100%)	1 (100%)
2009: N enrolled	55	8	19	20	5	3
Number of LSMs[(a)]	2 (2–4)	2 (2–3)	2 (2–3.5)	3 (2.5–4)	2 (2–2)	2 (2–2.5)
Age in years[(a)]	53 (44–56.5)	47.50 (42–52)	47 (40–55.5)	54.5 (52–61)	53 (50–57)	60 (57–61)
Sex M[(b)]	43 (78.2%)	6 (75.0%)	12 (63.2%)	17 (85.0%)	5 (100%)	3 (100%)
2010: N enrolled	56	10	20	21	3	2
Number of LSMs[(a)]	2.5 (2–4)	2.5 (2–4)	2 (2–3.5)	3 (2–4)	2 (2–2.5)	3 (2–4)
Age in years[(a)]	54 (46–58.5)	56.5 (50–61)	50.5 (44.5–56.5)	55 (46–59)	54 (50–56)	48 (41–55)
Sex M[(b)]	42 (75.0%)	5 (50.0%)	14 (70.0%)	18 (85.7%)	3 (100%)	3 (100%)

2011: N enrolled	40	3	18	17	–	2
Number of LSMs[(a)]	2.5 (2–4)	3 (2.5–3.5)	2 (2–4)	3 (2–4)	–	2.5 (2–3)
Age in years[(a)]	55 (50–57.5)	54 (52–57.5)	55 (47–58)	53 (50–56)	–	57
Sex M[(b)]	26 (65.0%)	2 (66.7%)	5 (27.8%)	17 (100%)	–	2 (100%)
2012: N enrolled	44	21	10	9	2	2
Number of LSMs[(a)]	2 (2–2)	2 (2–2)	2 (2–3)	2 (2–2)	2	3
Age in years[(a)]	54 (50–59)	57(51–60)	53.5 (42–62)	54(50–56)	52 (51–53)	49 (44–44)
Sex M[(b)]	29 (65.9%)	11 (52.4%)	5 (50.0%)	9 (100%)	2 (100%)	2 (100%)
2013: N enrolled	42	14	16	10	1	1
Number of LSMs[(a)]	2 (2–3)	2 (2–2)	3 (2–4)	2 (2–3)	3	2
Age in years[(a)]	57 (48–61)	54.5 (45–59)	56.5 (46–61.5)	58 (54–62)	59	76
Sex M[(b)]	25 (59.5%)	9 (64.3%)	5 (31.2%)	10 (100%)	1 (100%)	1 (100%)
2014: N enrolled	50	21	13	15	1	–
Number of LSMs[(a)]	2 (2–3)	2 (2–3)	2 (2–3)	3 (2–3)	2	–
Age in years[(a)]	56.5 (53–60)	58 (55–61)	53 (43–55)	58 (542.50–61.5)	55	–
Sex M[(b)]	34 (68.0%)	11 (52.4%)	9 (69.2%)	14 (93.3%)	–	–
2015: N enrolled	45	14	19	8	3	1
Number of LSMs[(a)]	2 (2–2)	2 (2–2)	2 (2–2)	2.5 (2–3.5)	2	3
Age in years[(a)]	58 (48–62)	58 (48–62)	58 (52–61.5)	58 (47.5–67)	65 (49.5–65)	56

Sex M(b)	26 (57.8%)	7 (50.0%)	7 (36.8%)	8 (100%)	3(100%)	1 (100%)
2016: N enrolled	45	11	16	17	1	–
Number of LSMs(a)	2 (2–2)	2 (2–3)	2 (2–2.5)	2 (2–2)	2	–
Age in years(a)	60 (54–65)	57 (51–59.5)	59.5 (51.5–63)	64 (56–66)	36	–
Sex M(b)	30 (66.7%)	6 (54.5%)	8 (50.0%)	15 (88.2%)	1 (100%)	–
2017: N enrolled	41	8	16	12	4	1
Number of LSMs(a)	2 (2–2)	2 (2–2)	2 (2–2)	2 (2–3)	2 (2–2.5)	2
Age in years(a)	60 (54–64)	55.5 (49.5–61.5)	61.5 (56.5–65.5)	58 (52–61)	57 (55–62)	71
Sex M(b)	27 (65.9%)	4 (50.0%)	7 (43.8%)	11 (91.7%)	4 (100%)	1 (100%)
2018: N enrolled	30	12	5	13	–	–
Number of LSMs(a)	2	2	2	2	–	–
Age in years(a)	58.5 (54–63)	61 (58.5–63.5)	53 (44–58)	64 (59–70.5)	–	–
Sex M(b)	18 (60.0%)	6 (50.0%)	2 (40.0%)	10 (76.9%)	–	–
2019: N enrolled	–	–	–	–	–	–

(a) either median (IQR) or the value when it was constant; (b) N (%)
